# Determination of the optimal sample size for a clinical trial accounting for the population size

**DOI:** 10.1002/bimj.201500228

**Published:** 2016-05-17

**Authors:** Nigel Stallard, Frank Miller, Simon Day, Siew Wan Hee, Jason Madan, Sarah Zohar, Martin Posch

**Affiliations:** ^1^ Statistics and Epidemiology, Division of Health Sciences, Warwick Medical School University of Warwick Coventry CV4 7AL UK; ^2^ Department of Statistics Stockholm University Stockholm Sweden; ^3^ Clinical Trials Consulting and Training Limited Buckingham UK; ^4^ Clinical Trials Unit, Warwick Medical School University of Warwick Coventry UK; ^5^ INSERM, U1138, team 22, Centre de Recherche des Cordeliers, Université Paris 5 Université Paris 6 Paris France; ^6^ Section of Medical Statistics, CeMSIIS Medical University of Vienna Austria

**Keywords:** Bayesian, Clinical trial design, Decision theory, Exponential family form, Optimal sample size

## Abstract

The problem of choosing a sample size for a clinical trial is a very common one. In some settings, such as rare diseases or other small populations, the large sample sizes usually associated with the standard frequentist approach may be infeasible, suggesting that the sample size chosen should reflect the size of the population under consideration. Incorporation of the population size is possible in a decision‐theoretic approach either explicitly by assuming that the population size is fixed and known, or implicitly through geometric discounting of the gain from future patients reflecting the expected population size. This paper develops such approaches. Building on previous work, an asymptotic expression is derived for the sample size for single and two‐arm clinical trials in the general case of a clinical trial with a primary endpoint with a distribution of one parameter exponential family form that optimizes a utility function that quantifies the cost and gain per patient as a continuous function of this parameter. It is shown that as the size of the population, *N*, or expected size, $N^{*}$ in the case of geometric discounting, becomes large, the optimal trial size is $O(N^{1/2})$ or $O(N^{*1/2})$. The sample size obtained from the asymptotic expression is also compared with the exact optimal sample size in examples with responses with Bernoulli and Poisson distributions, showing that the asymptotic approximations can also be reasonable in relatively small sample sizes.

## 1 Introduction

The problem of determining the sample size for a clinical trial is a very common one. For large‐scale definitive phase III clinical trials, a frequentist approach is usually adopted, with the sample size chosen so as to control the type I error rate at a specified level, α, and to give specified power 1−β, to detect some appropriately chosen size of treatment effect (see, e.g. Pocock, 1983, for details). Choice of α=0.05 and β=0.1 or 0.2 is typical.

The sample sizes obtained using the frequentist approach do not always seem appropriate. In particular they do not reflect the size of the population to which the results of the trial apply. The population size is relevant, however, when considering decisions made on the basis of trial results. This is particularly true for clinical trials conducted in rare diseases or other small populations, when the population size means that a large trial would be infeasible, or even impossible.

One way in which the size of the population can influence the sample size is to use a decision theoretic approach in which the benefits to future patients in the population, sometimes called the “patient horizon”, are explicitly considered so that future benefit depends on the size of this population. Such an approach has been proposed and discussed by numerous authors over the last 50 years (see, e.g. Anscombe, 1963; Colton, 1963; Sylvester, 1988; Berry et al., 1994; Cheng et al., 2003; Kikuchi and Gittins, 2009 and reviews by Pezeshk et al., 2013; Hee et al., 2016). Although this approach has very rarely been implemented in practice, it can nevertheless provide important insight into an appropriate choice of sample size for a clinical trial.

The role of the population size in determination of the optimal sample size for a clinical trial has been considered by Cheng et al. (2003). They considered single‐arm and two‐arm clinical trials with a primary endpoint following a Bernoulli distribution indicating either success or failure of treatment for each patient. Adopting a decision‐theoretic approach, they obtained designs that maximize the total number of successes. Denoting the population size by *N*, they show that asymptotically as N→∞, the optimal sample size for a clinical trial is $O(N^{1/2})$ and give an expression for the asymptotically optimal sample size that depends on the prior distributions for the unknown probability of success for each trial arm.

In this paper, we extend the work of Cheng et al. (2003), both to more general distributional forms for the primary endpoint and to situations in which the aim is to maximize some general utility expressed as a function of a parameter of these distributions. We show that the result that the optimal sample size is $O(N^{1/2})$ applies for any continuous utility function and for responses with a distribution of any one‐parameter exponential family form assuming a conjugate prior distribution. We also consider the case where no finite patient horizon is assumed, but gains from future patients are geometrically discounted, that is the gain from the *j*th patient is multiplied by $\lambda^{j-1}$ for some discounting parameter, λ<1 (Berry and Fristedt, 1985). As considered in the discussion section, the size of λ can also reflect the size of the patient population in this setting, via an effective number of patients, ${\left(1-\lambda \right)}^{-1}$, which will be denoted $N^{*}$. We show that in this case as λ→1 so that $N^{*}$ is large, the optimal sample size is $O(N^{*1/2})$. We also investigate through exact calculation the small‐sample accuracy of the the large sample approximations. Although the results obtained depend on asymptotics, we show that, depending on the exact form of the utility function chosen, these may be reasonable even for extremely rare diseases, for example for patient populations of 1000 or less when the optimal sample size can be less than 50.

## 2 Detailed problem description and notation

### 2.1 Outline of the decision problem

Suppose that a clinical trial is to be conducted to choose between two treatments with *n*_1_ and *n*_2_ patients receiving treatment 1 and treatment 2, respectively, where treatment 2 may be the current standard treatment included as a control. Note that taking $n_{2}=0$ corresponds to a single‐arm trial, though the decision to be taken at the end of such a trial remains comparative, with a choice being made regarding treatment of future patients.

It is assumed that the gain associated with treatment of patients in the trial or patients outside the trial who receive each treatment can be specified as a function of a parameter of the distribution for the response for patients receiving that treatment. It is noted that here “gain” is to be interpreted widely to include any kind of costs, losses, gains, or benefits associated with treatment. Following the trial, the most preferable treatment, that is the treatment for which the posterior expected gain given the observed data, is highest, will be selected. The remaining patients will then receive this treatment. We wish to determine the optimal values, $n_{1}^{*}$ and $n_{2}^{*}$, of the sample sizes *n*_1_ and *n*_2_ and to determine how $n_{1}^{*}$ and $n_{2}^{*}$ depend on the population size.

### 2.2 Decision problem formulation and notation

We will assume that responses for patients follow some distribution of natural one‐parameter exponential family form. In detail, let $Y_{ij}$ denote the response for patient *j* receiving treatment *i* and assume $Y_{i1},\ldots ,Y_{in_{i}}$ are i.i.d. with density $f_{i}\left(y|\psi_{i}\right)=a_{i}\left(y\right)\exp\left(y\psi_{i}-b_{i}\left(\psi_{i}\right)\right)$ for some $a_{i}\left(y\right)$ and $b_{i}\left(\psi_{i}\right)$. Typically responses from patients in the two treatment groups will follow distributions of the same form, that is functions $a_{i}$ and $b_{i}$ will not depend on *i*, with ψ_1_ and ψ_2_ differing, though this is not assumed. We will assume that ψ_1_ and ψ_2_ are taken to have independent prior distributions of conjugate form, that is with $\psi_{i}$ having density $\pi_{i}\left(\psi_{i}|n_{0i},y_{0i}\right)=c_{i}\left(n_{0i},y_{0i}\right)\exp\left(n_{0i}y_{0i}\psi_{i}-n_{0i}b_{i}\left(\psi_{i}\right)\right)$ for some $y_{0i}$ and $n_{0i}$ and normalising constant $c_{i}\left(n_{0i},y_{0i}\right),i=1,2$. The values $y_{0i}$ and $n_{0i}$ can be interpreted respectively as the prior mean of $\xi_{i}=b_{i}^{\prime }\left(\psi_{i}\right)=E\left(Y_{ij}\right)$ and the number of observations to which the prior information is equivalent, so that following a trial with $n_{i}$ patients receiving treatment and observation of $Y=(Y_{11},\ldots ,Y_{1n_{1}},Y_{21},\ldots ,Y_{2n_{2}})$, the posterior mean for $\xi_{i}$ given Y is equal to

(1) $$\frac{n_{0i}y_{0i}+n_{i}{\bar{Y}}_{i}}{n_{0i}+n_{i}},i=1,2$$

with ${\bar{Y}}_{i}=\sum_{j=1}^{n_{i}}Y_{ij}/n_{i}$ (see Bernado and Smith, 2000).

Suppose that the expected gain from a patient receiving treatment *i* in the trial is $h_{i}\left(\xi_{i}\right)$, and that the expected gain to a future patient receiving treatment *i* is $g_{i}\left(\xi_{i}\right)$ where $h_{i}$ and $g_{i},i=1,2$ are such that the expected values $E_{0}\left(h_{i}\left(\xi_{i}\right)\right)$, $E_{0}\left(g_{i}\left(\xi_{i}\right)\right)$ and $E_{0}\left(\max_{i=1,2}\left(g_{i}\left(\xi_{i}\right)\right)\right)$ where *E*_0_ denotes the expected value taken over the prior distribution of ξ, exist and $h_{i}$ and $g_{i}$ are assumed to be differentiable with $g_{i}$ strictly increasing and with finite derivative. Assume further that

(2) $$E_{0}\left(h_{i}\left(\xi_{i}\right)\right)\leq E_{0}\left(\max\left\{g_{1}\left(\xi_{1}\right),g_{2}\left(\xi_{2}\right)\right\}\right),i=1,2.$$

This ensures that the gain from treating patients in the trial cannot exceed that from treating them outside the trial. This is considered further in the discussion section below.

We will consider two cases. In the first, the population is considered to be finite with known size, *N*. The number of patients treated following the trial is thus $N-n_{1}-n_{2}$. In the second case, no finite population size is assumed, but the gain from future patients is geometrically discounted, so that the gain from patient *j* if they receive treatment *i* is $\lambda^{j-1}h_{i}\left(\xi_{i}\right)$ if they are included in the trial and $\lambda^{j-1}g_{i}\left(\xi_{i}\right)$ if they are treated following the trial, for some λ<1.

The geometric discounting of gains from future patients can be interpreted in a number of ways. One interpretation is that gains further in the future are reduced to reflect either opportunity loss or loss of financial interest on an investment (see, e.g. Fergusson, 2008). With this interpretation it might be appropriate to take λ<1 and *N* finite. An alternative interpretation is to imagine that gain from each future patient is of constant value, as is assumed in the finite horizon case, but that the size of the population, *N*, rather than being fixed in advance, is random, following a geometric distribution. This could be the case if, for example, the population is limited by some new treatment becoming available at which point the trial, or use of the recommended treatment following the trial, will be terminated, with the probability of this event constant over time (see Berry and Fristedt, 1985). In this interpretation $N^{*}={\left(1-\lambda \right)}^{-1}$ is the expected population size prior to this new treatment becoming available. The size of λ and the resulting $N^{*}$ can thus reflect the population size, and in some ways a smaller value of λ, corresponding to fewer patients being available prior to the new treatment becoming available, more reasonably models a small population than assuming the number of patients has some fixed and known value, *N*.

## 3 Determination of the optimal sample size

### 3.1 Finite patient horizon case

Consider first the setting of a finite patient horizon of size *N*. Following observation of data Y= $\left(Y_{11},\ldots ,Y_{1n_{1}},Y_{21},\ldots ,Y_{2n_{2}}\right)$, the total expected gain if treatment *i* is recommended for all further $N-n_{1}-n_{2}$ patients is $n_{1}E_{\xi |Y}\left(h_{1}\left(\xi_{1}\right)|Y\right)+n_{2}(E_{\xi |Y}\left(h_{2}\left(\xi_{2}\right)|Y\right)+\left(N-n_{1}-n_{2}\right)E_{\xi |Y}\left(g_{i}\left(\xi_{i}\right)|Y\right)$ where $E_{\xi |Y}\left(.|Y\right)$ denotes the expected value taken over the posterior distribution of ξ given Y.

The optimal action at the end of the trial is thus to select the treatment with the largest value of $E_{\xi |Y}\left(g_{i}\left(\xi_{i}\right)|Y\right)$ and the expected gain assuming this action is taken is equal to $n_{1}E_{\xi |Y}\left(h_{1}\left(\xi_{1}\right)|Y\right)+n_{2}E_{\xi |Y}\left(h_{2}\left(\xi_{2}\right)|Y\right)+\left(N-n_{1}-n_{2}\right)\max_{i=1,2}E_{\xi |Y}\left(g_{i}\left(\xi_{i}\right)|Y\right).$

Since, prior to the commencement of the trial, Y is unknown, the expected gain from the trial is equal to $E_{0}\left(G\right)$ where the expectation is taken over the prior distribution for ξ_1_ and ξ_2_ and G is the function of ξ_1_, ξ_2_, $N,n_{1}$, and *n*_2_ given by

(3) $$\begin{array}{ccc} G & = & n_{1}E_{Y}\left(E_{\xi |Y}\left(h_{1}\left(\xi_{1}\right)|Y\right)\right)+n_{2}E_{Y}\left(E_{\xi |Y}\left(h_{2}\left(\xi_{2}\right)|Y\right)\right)+\\ & & +\left(N-n_{1}-n_{2}\right)E_{Y}\left(\max_{i=1,2}E_{\xi |Y}\left(g_{i}\left(\xi_{i}\right)|Y\right)\right) \end{array}$$

where $E_{Y}$ denotes the expectation taken over Y for a given value of ξ_1_ and ξ_2_ so that expectations are taken first over the posterior distributions of $\xi_{i}$ given Y and then over Y given ξ_1_ and ξ_2_.

Since $E_{0}\left(E_{Y}\left(E_{\xi |Y}\left(h_{i}\left(\xi_{i}\right)|Y\right)\right)\right)$ is equal to the prior expectation $E(h_{i}\left(\xi_{i}\right))$ for any function $h_{i}$ for which the expectations exist, we get

(4) $$E_{0}\left(G\right)=n_{1}E_{0}\left(h_{1}\left(\xi_{1}\right)\right)+n_{2}E_{0}\left(h_{2}\left(\xi_{2}\right)\right)+\left(N-n_{1}-n_{2}\right)E_{0}\left(E_{Y}\left(\max_{i=1,2}E_{\xi |Y}\left(g_{i}\left(\xi_{i}\right)|Y\right)\right)\right).$$

We wish to find the optimal values of *n*_1_ and *n*_2_, that is the values for which $E_{0}\left(G\right)$ is maximized. For small *N*, when the optimal sample sizes, $n_{1}^{*}$ and $n_{2}^{*}$ will also be small, it may be feasible to evaluate the prior expected gain given by (4) directly, taking the expectation over the prior predictive distribution for Y and to find $n_{1}^{*}$ and $n_{2}^{*}$ by a numerical search. For larger *N*, such an approach may be infeasible. In this case asymptotic expressions for the optimal sample sizes, $n_{1}^{*}$ and $n_{2}^{*}$, as the population size, *N*, becomes large are more useful.

For finite *n*_01_ and *n*_02_, the expectation $E_{0}\left(E_{Y}\left(\max_{i=1,2}E_{\xi |Y}\left(g_{i}\left(\xi_{i}\right)|Y\right)\right)\right)$ is increasing in *n*_1_ and *n*_2_. Thus for $N>n_{1}-n_{2}$, $\left(N-n_{1}-n_{2}\right)E_{0}\left(E_{Y}\left(\max_{i=1,2}E_{\xi |Y}\left(g_{i}\left(\xi_{i}\right)|Y\right)\right)\right)$ and hence $E_{0}\left(G\right)$ is also increasing in *n*_1_ and *n*_2_. Thus as N→∞ the optimal trial design has both $(n_{01}+n_{1})\to \infty$ and $(n_{02}+n_{2})\to \infty$. The case in which both *n*_01_ and *n*_02_ are both infinite corresponds to both ξ_1_ and ξ_2_ being known *a priori*. We will therefore consider the case in which, without loss of generality, *n*_01_ is finite, and derive the optimal value $n_{1}^{*}$.

Note that as N→∞, so that the optimal $n_{01}+n_{1}$ and $n_{02}+n_{2}$ also approach infinity, $E_{0}\left(E_{Y}\left(\max_{i=1,2}E_{\xi |Y}\left(g_{i}\left(\xi_{i}\right)|Y\right)\right)\right)\to \max_{i=1,2}E_{0}\left(g_{i}\left(\xi_{i}\right)\right)$ so that the optimal sample sizes are such that $n_{i}/N\to 0,i=1,2$.

By the central limit theorem we have ${\bar{Y}}_{i}\overset{d}{\to }N\left(\xi_{i},v_{i}\left(\xi_{i}\right)/n_{i}\right)$ where $v_{i}\left(\xi_{i}\right)$ denotes the variance of $Y_{ij}$. Thus from (1), applying the delta method since $g_{i}$ is assumed to be differentiable and strictly increasing so that the derivative, $g_{i}^{\prime }\left(\xi_{i}\right)$, is non‐zero, we get

(5) $$E_{\xi |Y}\left(g_{i}\left(\xi_{i}\right)|{\bar{Y}}_{i}\right)\overset{d}{\to }N\left(g_{i}\left(\frac{n_{0i}y_{0i}+n_{i}\xi_{i}}{n_{0i}+n_{i}}\right),{\left(\frac{n_{i}}{n_{0i}+n_{i}}\right)}^{2}{\left(g_{i}^{\prime }\left(\xi_{i}\right)\right)}^{2}\frac{v_{i}\left(\xi_{i}\right)}{n_{i}}\right).$$

Using an expression for the expected value of the maximum of two normally distributed random variables given by Clark (1961), we have

(6) $$E_{Y}\left(\max_{i=1,2}E_{\xi |Y}\left(g_{i}\left(\xi_{i}\right)|Y\right)\right)\to {\tilde{\xi }}_{1}\Phi \left(\delta \right)+{\tilde{\xi }}_{2}\Phi \left(-\delta \right)+a\phi \left(\delta \right)$$

where ${\tilde{\xi }}_{i}=g_{i}\left(\left(n_{0i}y_{0i}+n_{i}\xi_{i}\right)/\left(n_{0i}+n_{i}\right)\right)$, $a^{2}=\sum_{i=1}^{2}n_{i}^{2}{\left(g_{i}^{\prime }\left(\xi_{i}\right)\right)}^{2}v_{i}\left(\xi_{i}\right)/n_{i}{\left(n_{0i}+n_{i}\right)}^{2}$, $\delta =({\tilde{\xi }}_{1}-{\tilde{\xi }}_{2})/a$ and ϕ and Φ denote standard normal density and distribution functions. Thus

(7) $$\begin{array}{c} E_{0}\left(G\right)\to n_{1}E_{0}\left(h_{1}\left(\xi_{1}\right)\right)+n_{2}E_{0}\left(h_{2}\left(\xi_{2}\right)\right)+\left(N-n_{1}-n_{2}\right)E_{0}\left({\tilde{\xi }}_{1}\Phi \left(\delta \right)+{\tilde{\xi }}_{2}\Phi \left(-\delta \right)+a\phi \left(\delta \right)\right). \end{array}$$

In order to find $n_{1}^{*}$, we obtain the derivative $\partial E_{0}\left(G\right)/\partial n_{1}=E_{0}\left(\partial G/\partial n_{1}\right)$. From (3),

(8) $$\begin{array}{c} \frac{\partial G}{\partial n_{1}}=h_{1}\left(\xi_{1}\right)-E_{Y}\left(\max_{i=1,2}E_{\xi |Y}\left(g_{i}\left(\xi_{i}\right)|Y\right)\right)+\left(N-n_{1}-n_{2}\right)\frac{\partial E_{Y}\left(\max_{i=1,2}E_{\xi |Y}\left(g_{i}\left(\xi_{i}\right)|Y\right)\right)}{\partial n_{1}}. \end{array}$$

We will thus find a large‐sample approximation for this derivative.

Equation (6) gives an approximation for $E_{Y}\left(\max_{i=1,2}E_{\xi |Y}\left(g_{i}\left(\xi_{i}\right)|Y\right)\right)$. The derivative of the right hand side of (6) is equal to

$$\begin{array}{c} \Phi \left(\delta \right)\frac{g_{1}^{\prime }\left(\xi_{1}\right)n_{01}\left(\xi_{i}-y_{01}\right)}{{\left(n_{01}+n_{1}\right)}^{2}}+\phi \left(\delta \right)\frac{\left(n_{01}-n_{1}\right)v_{1}\left(\xi_{1}\right){\left(g_{i}^{\prime }\left(\xi_{i}\right)\right)}^{2}}{2a{\left(n_{01}+n_{1}\right)}^{3}} \end{array}$$

(see Web Appendix A for details), so that $\partial G/\partial n_{1}$ can be approximated by

$$\begin{array}{ccc} h_{1}\left(\xi_{1}\right) & - & E_{Y}\left(\max_{i=1,2}E_{\xi |Y}\left(g_{i}\left(\xi_{i}\right)|Y\right)\right)+\\ & & +\left(N-n_{1}-n_{2}\right)\left(\Phi \left(\delta \right)\frac{g_{1}^{\prime }\left(\xi_{1}\right)n_{01}\left(\xi_{i}-y_{01}\right)}{{\left(n_{01}+n_{1}\right)}^{2}}+\phi \left(\delta \right)\frac{\left(n_{01}-n_{1}\right)v_{1}\left(\xi_{1}\right){\left(g_{i}^{\prime }\left(\xi_{i}\right)\right)}^{2}}{2a{\left(n_{01}+n_{1}\right)}^{3}}\right). \end{array}$$

The limit of this as *N*, *n*_1_ and $n_{02}+n_{2}$ all approach infinity with $n_{1}/N$ and $n_{2}/N$ approaching 0 (again, see Web Appendix A for details), has expected value

(9) $$\begin{array}{c} E_{0}\left(h_{1}\left(\xi_{1}\right)-\max_{i=1,2}g_{i}\left(\xi_{i}\right)\right)+\frac{N}{n_{1}^{2}}\int -\frac{v_{1}\left(g_{1}^{-1}\left(g_{2}\left(\xi_{2}\right)\right)\right)}{2}\left(g_{1}^{\prime }\left(g_{1}^{-1}\left(g_{2}\left(\xi_{2}\right)\right)\right)\right)\pi \left(g_{1}^{-1}\left(g_{2}\left(\xi_{2}\right)\right),\xi_{2}\right)d\xi_{2} \end{array}$$

where $\pi (\xi_{1},\xi_{2})$ denotes the joint prior distribution of ξ_1_ and ξ_2_.

Setting this to zero and solving for *n*_1_, the maximum expected gain is found to be at

(10) $$n_{1}^{*}=\sqrt{\frac{N\int v_{1}\left(g_{1}^{-1}\left(g_{2}\left(\xi_{2}\right)\right)\right)g_{1}^{\prime }\left(g_{1}^{-1}\left(g_{2}\left(\xi_{2}\right)\right)\right)\pi \left(g_{1}^{-1}\left(g_{2}\left(\xi_{2}\right)\right),\xi_{2}\right)d\xi_{2}}{2(E_{0}\left(\max_{i=1,2}g_{i}\left(\xi_{i}\right)\right)-E_{0}\left(h_{1}\left(\xi_{1}\right)\right))}}.$$

Note that the fact that *g*_1_ is increasing and the requirement (2) ensures that both numerator and denominator are positive so that the square root exists. It is also interesting to note that the asymptotic optimal sample size for arm 1, *n*_1_, does not depend on *n*_2_ since we have assumed either that *n*_02_ is infinite or that *n*_2_ also approaches infinity.

When *n*_02_ is finite, by symmetry, the optimal value of *n*_2_ is given by

(11) $$n_{2}^{*}=\sqrt{\frac{N\int v_{2}\left(g_{2}^{-1}\left(g_{1}\left(\xi_{1}\right)\right)\right)g_{2}^{\prime }\left(g_{2}^{-1}\left(g_{1}\left(\xi_{1}\right)\right)\right)\pi \left(\xi_{1},g_{2}^{-1}\left(g_{1}\left(\xi_{1}\right)\right)\right)d\xi_{1}}{2(E_{0}\left(\max_{i=1,2}g_{i}\left(\xi_{i}\right)\right)-E_{0}\left(h_{2}\left(\xi_{2}\right)\right))}}.$$

When $n_{02}=\infty$, the prior distribution has mass at $\xi_{2}=y_{02}$ only, and so may be written as a univariate density $\pi (\xi_{1})$, so that the optimal value of *n*_1_ becomes

(12) $$n_{1}^{*}=\sqrt{\frac{Nv_{1}\left(g_{1}^{-1}\left(g_{2}\left(y_{02}\right)\right)\right)g_{1}^{\prime }\left(g_{1}^{-1}\left(g_{2}\left(y_{02}\right)\right)\right)\pi \left(g_{1}^{-1}\left(g_{2}\left(y_{02}\right)\right)\right)}{2(E_{0}\left(\max_{i=1,2}g_{i}\left(\xi_{i}\right)\right)-E_{0}\left(h_{1}\left(\xi_{1}\right)\right))}}.$$

As $n_{02}\to \infty$, $\partial E_{Y}\left(\max_{i=1,2}E_{\xi |Y}\left(g_{i}\left(\xi_{i}\right)|Y\right)\right)/\partial n_{2}\to 0$. Thus from an expression similar to (8) giving the derivative with respect to *n*_2_ and (2), the derivative is negative and $n_{2}^{*}=0$.

When *g*_1_ and *g*_2_ are identical, (10) becomes

$$\begin{array}{c} n_{1}^{*}=\sqrt{\frac{N\int v_{1}\left(\xi_{2}\right)g_{1}^{\prime }\left(\xi_{2}\right)\pi \left(\xi_{2},\xi_{2}\right)d\xi_{2}}{2(E_{0}\left(\max_{i=1,2}g_{i}\left(\xi_{i}\right)\right)-E_{0}\left(h_{1}\left(\xi_{1}\right)\right))}}. \end{array}$$

and when $g_{i}\left(\xi_{i}\right)=h_{i}\left(\xi_{i}\right)=\xi_{i},i=1,2$, this becomes

$$\begin{array}{c} n_{1}^{*}=\sqrt{\frac{N\int v_{1}\left(\xi_{2}\right)\pi \left(\xi_{2},\xi_{2}\right)d\xi_{2}}{2(E_{0}\left(\max_{i=1,2}\xi_{i}\right)-E_{0}\left(\xi_{1}\right))}} \end{array}$$

showing that this is a generalization of the expression obtained by Cheng et al. (2003) for the case in which $Y_{ij}$ has a Bernoulli distribution with parameter $\xi_{i}$ and $v_{i}\left(\xi_{i}\right)=\xi_{i}\left(1-\xi_{i}\right)$.

### 3.2 Geometric discounting case

Consider next the second setting introduced above; that of an infinite population with geometric discounting.

In a two‐arm trial it is assumed that *n*_1_ and *n*_2_ are sufficiently large and randomisation to treatments 1 and 2 sufficiently balanced that the gain to patients receiving treatment *i* in the trial can be taken to be $n_{i}\sum_{j=1}^{n_{1}+n_{2}}\lambda^{j-1}/\left(n_{1}+n_{2}\right)h_{i}\left(\xi_{i}\right)$. The total expected gain if treatment *i* is recommended for all further $N-n_{1}-n_{2}$ patients is then

$$\begin{array}{ccc} \frac{n_{1}}{n_{1}+n_{2}}\sum_{j=1}^{n_{1}+n_{2}}\lambda^{j-1}E_{\xi |Y}\left(h_{1}\left(\xi_{1}\right)|Y\right) & + & \frac{n_{2}}{n_{1}+n_{2}}\sum_{j=1}^{n_{1}+n_{2}}\lambda^{j-1}E_{\xi |Y}\left(h_{2}\left(\xi_{2}\right)|Y\right)+\\ & & +\;\sum_{j=n_{1}+n_{2}+1}^{\infty }\lambda^{j-1}E_{\xi |Y}\left(g_{i}\left(\xi_{i}\right)|Y\right). \end{array}$$

The optimal action at the end of the trial is thus again to treat all future patients with the treatment with the largest value of $E_{\xi |Y}\left(g_{i}\left(\xi_{i}\right)|Y\right)$ and the expected gain assuming this action is taken is equal to

$$\begin{array}{ccc} \frac{n_{1}}{n_{1}+n_{2}}\sum_{j=1}^{n_{1}+n_{2}}\lambda^{j-1}E_{\xi |Y}\left(h_{1}\left(\xi_{1}\right)|Y\right) & + & \frac{n_{2}}{n_{1}+n_{2}}\sum_{j=1}^{n_{1}+n_{2}}\lambda^{j-1}E_{\xi |Y}\left(h_{2}\left(\xi_{2}\right)|Y\right)+\\ & & +\sum_{j=n_{1}+n_{2}+1}^{\infty }\lambda^{j-1}\max_{i=1,2}E_{\xi |Y}\left(g_{i}\left(\xi_{i}\right)|Y\right). \end{array}$$

The prior predicted expected utility for a trial with $n_{i}$ patients receiving treatment *i* is thus

(13) $$\begin{array}{ccc} \frac{n_{1}}{n_{1}+n_{2}}\sum_{j=1}^{n_{1}+n_{2}}\lambda^{j-1}E_{0}\left(h_{1}\left(\xi_{1}\right)\right) & + & \frac{n_{2}}{n_{1}+n_{2}}\sum_{j=1}^{n_{1}+n_{2}}\lambda^{j-1}E_{0}\left(h_{2}\left(\xi_{2}\right)\right)+\\ & & +\sum_{j=n_{1}+n_{2}+1}^{\infty }\lambda^{j-1}E_{0}\left(E_{Y}\left(\max_{i=1,2}E_{\xi |Y}\left(g_{i}\left(\xi_{i}\right)|Y\right)\right)\right). \end{array}$$

Optimal sample sizes, $n_{1}^{*}$ and $n_{2}^{*}$, can be found directly using this expression and a numerical search in cases when this is computationally feasible.

As above, it is of interest to seek asymptotic approximations to $n_{1}^{*}$ and $n_{2}^{*}$, in this case as the geometric discounting parameter, λ, approaches 1 from below. We will again assume that *n*_01_ is finite and obtain first an approximation for $n_{1}^{*}$.

The derivatives of $\sum_{j=1}^{n_{1}+n_{2}}\lambda^{j-1}=\left(1-\lambda^{n_{1}+n_{2}}\right)/\left(1-\lambda \right)$ and $\sum_{j=n_{1}+n_{2}+1}^{\infty }\lambda^{j-1}=\lambda^{n_{1}+n_{2}}/\left(1-\lambda \right)$ with respect to *n*_1_ are respectively $-\lambda^{n_{1}+n_{2}}\log\lambda /\left(1-\lambda \right)$ and $\lambda^{n_{1}+n_{2}+1}\log\lambda /\left(1-\lambda \right)$ which, by L'Hospital's rule, tend to 1 and −1, respectively as λ→1, so that, since the limit as λ→1 of $\sum_{j=1}^{n_{1}+n_{2}}\lambda^{j-1}$ is $n_{1}+n_{2}$, the derivative of the term $n_{i}\sum_{j=1}^{n_{1}+n_{2}}\lambda^{j-1}/\left(n_{1}+n_{2}\right)$ with respect to *n*_1_ tends to 1 if i=1 and 0 if i=2.

As λ→1, the derivative of (13) with respect to *n*_1_ thus approaches

$$\begin{array}{c} h_{1}\left(\xi_{1}\right)-E_{Y}\left(\max_{i=1,2}E_{\xi |Y}\left(g_{i}\left(\xi_{i}\right)|Y\right)\right)+\left(\sum_{j=n_{1}+n_{2}+1}^{\infty }\lambda^{j-1}\right)\frac{\partial E_{Y}\left(\max_{i=1,2}E_{\xi |Y}\left(g_{i}\left(\xi_{i}\right)|Y\right)\right)}{\partial n_{1}}. \end{array}$$

The argument above gives an approximation to this derivative of

$$\begin{array}{ccc} h_{1}\left(\xi_{1}\right) & - & \max_{i=1,2}g_{i}\left(\xi_{i}\right)+\\ & & +\frac{\sum_{j=n_{1}+n_{2}+1}^{\infty }\lambda^{j-1}}{n_{1}^{2}}\int -\frac{v_{1}\left(g_{1}^{-1}\left(g_{2}\left(\xi_{2}\right)\right)\right)}{2}\left(g_{1}^{\prime }\left(g_{1}^{-1}\left(g_{2}\left(\xi_{2}\right)\right)\right)\right)\pi \left(g_{1}^{-1}\left(g_{2}\left(\xi_{2}\right)\right),\xi_{2}\right)d\xi_{2}, \end{array}$$

which, since $\sum_{j=n_{1}+n_{2}+1}^{\infty }\lambda^{j-1}=\sum_{j=1}^{\infty }\lambda^{j-1}-\sum_{j=1}^{n_{1}+n_{2}}\lambda^{j-1}$, with the former term, which is equal to ${\left(1-\lambda \right)}^{-1}$, dominating, gives the approximation

(14) $$n_{1}^{*}=\sqrt{\frac{\int v_{1}\left(g_{1}^{-1}\left(g_{2}\left(\xi_{2}\right)\right)\right)g_{1}^{\prime }\left(g_{1}^{-1}\left(g_{2}\left(\xi_{2}\right)\right)\right)\pi \left(g_{1}^{-1}\left(g_{2}\left(\xi_{2}\right)\right),\xi_{2}\right)d\xi_{2}}{2\left(1-\lambda \right)\left(E_{0}\left(\max_{i=1,2}g_{i}\left(\xi_{i}\right)\right)-E_{0}\left(h_{1}\left(\xi_{1}\right)\right)\right)}}.$$

Writing $N^{*}=\sum_{j=1}^{\infty }\lambda^{j-1}={\left(1-\lambda \right)}^{-1}$, (14) can be written as

$$\begin{array}{c} n_{1}^{*}=\sqrt{\frac{N^{*}\int v_{1}\left(g_{1}^{-1}\left(g_{2}\left(\xi_{2}\right)\right)\right)g_{1}^{\prime }\left(g_{1}^{-1}\left(g_{2}\left(\xi_{2}\right)\right)\right)\pi \left(g_{1}^{-1}\left(g_{2}\left(\xi_{2}\right)\right),\xi_{2}\right)d\xi_{2}}{2\left(E_{0}\left(\max_{i=1,2}g_{i}\left(\xi_{i}\right)\right)-E_{0}\left(h_{1}\left(\xi_{1}\right)\right)\right)}}, \end{array}$$

directly analogous to (10) with $N^{*}$ replacing *N*.

For *n*_02_ finite, $n_{2}^{*}$ is again given by symmetry by an expression analogous to (11). For $n_{02}=\infty$, $n_{2}^{*}=0$ and $n_{1}^{*}$ is given by an expression analogous to (12) with *N* replaced by $N^{*}$.

## 4 Examples

### 4.1 Single arm trials with Bernoulli data

We consider first the case of Bernoulli data. In this case the distribution of $Y_{ij}$, the responses in treatment group *i*, can be parameterised with $\xi_{i}$ equal to the probability of treatment success. We will take ξ_1_ to have a conjugate beta prior with parameters *a*_1_ and *b*_1_ so that $y_{01}=a_{1}/\left(a_{1}+b_{1}\right)$ and $n_{01}=a_{1}+b_{1}$, and assume ξ_2_ is known with value *y*_02_, that is with $n_{02}=\infty$, so that $n_{2}^{*}=0$ and a single‐arm trial is optimal.

Given observation of data $Y_{1j}=y_{1j},j=1,\ldots ,n_{1}$, ξ_1_ has a Beta$\left(a_{1}+\sum_{j=1}^{n_{1}}y_{1j},b_{1}+n_{1}-\sum_{j=1}^{n_{1}}y_{1j}\right)$ posterior distribution, and the prior predictive distribution of $\sum_{j=1}^{n_{1}}Y_{1j}$ is Betabinomial(*n*_1_, *a*_1_, *b*_1_).

We will assume that the gain from patients receiving treatment *i* will be determined by whether or not the treatment is successful, so that $g_{i}\left(\xi_{i}\right)=h_{i}\left(\xi_{i}\right)=\xi_{i},i=1,2$. From (4) with $n_{2}=0$, the prior expected gain is thus $n_{1}E_{0}\left(\xi_{1}\right)+\left(N-n_{1}\right)E_{0}\left(E_{Y}\left(\max_{i=1,2}E_{\xi |Y}\left(\xi_{i}\right)|Y\right)\right).$

**Figure 1:**
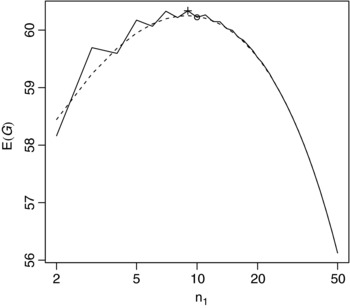
Prior expected gain (solid line) and approximate prior expected gain (dashed line) for a range of *n*_1_ for the first single‐arm Bernoulli example with $a_{1}=b_{1}=1$, $\xi_{2}=0.5$, and N=100. The optimal and approximately optimal values of *n*_1_ are marked by + and ○, respectively

Following one of the examples considered by Cheng et al. (2003), we take $a_{1}=1$ and $b_{1}=1$, the known value of ξ_2_ to be 0.5 and N=100. Figure 1 shows the prior expected gain for a range of values of *n*_1_, here plotted on a logarithmic scale. As given by Cheng et al. (2003), the optimal value of *n*_1_ is equal to 9, which is marked with a plus sign. The approximation to the prior expected gain given by (7) is also shown on the figure as a dashed line, showing that in this case the approximate and true values are close even for small *n*_1_. The approximately optimal value of *n*_1_ given by (12) is 10. This is shown by the circle on the figure, showing that this is close to the true optimum. In this case, this is close to the value of *n*_1_ maximizing the approximation given by (7), though in general the additional approximation leading to (12) means that this need not be the case.

**Figure 2:**
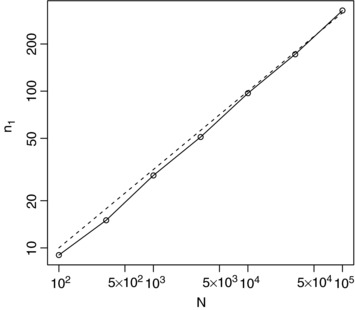
Optimal (solid lines with points) and approximately optimal (dashed lines) values for *n*_1_ for a range of *N* values for the first single‐arm Bernoulli example with $a_{1}=b_{1}=1$ and $\xi_{2}=0.5$.

Figure 2 gives values of $n_{1}^{*}$ from (12) along with exact optima for a range of values of *N*, with both $n_{1}^{*}$ and *N* plotted on logarithmic scales so that the square root relationship between $n_{1}^{*}$ and *N* given by (12) corresponds to a straight line with slope 1/2. The approximation given by (12) is close to the true value, and approaches it as *N* increases as would be expected.

The effect of varying the prior distribution for ξ_1_ was also investigated. Web Figure 1 in Web Appendix B shows the optimal sample size $n_{1}^{*}$ with N=100 and $\xi_{2}=0.5$ for a range of $n_{01}=a_{1}+b_{1}$ and $y_{01}=a_{1}/\left(a_{1}+b_{1}\right)$ values. When the prior mean value of ξ_1_, that is *y*_01_, is equal or greater than the fixed value of ξ_2_, the optimal sample size increases with prior weight *n*_01_. This is reasonable since as *n*_01_ increases we are increasingly confident that patients are not harmed by being in the trial and a larger sample size gives more information for the final decision. When *y*_01_ is less than ξ_2_, the optimal sample size increases with *n*_01_ for small *n*_01_, so that more information can be collected for the final decision and decreases for larger *n*_01_ when there is strong prior belief that treatment 2 is superior to treatment 1. It is interesting to note that when the prior weight, *n*_01_, is kept fixed, the optimal sample size increases as the prior mean *y*_01_ of treatment 1 increases. This is in contrast to frequentist sample size that would decrease as the assumed success rate for treatment 1 is increased (and is larger than the success rate of treatment 2).

We next consider an example based on Stallard (1998), who also considered a single‐arm phase II trial with a Bernoulli outcome. In this case the gain function was chosen to reflect the financial costs and rewards associated with the conduct of the trial assuming that, if successful, it would be followed by a further trial with a frequentist design and analysis. Assuming the probability of success for treatment 2, here taken to be the current standard treatment, to be known, costs and rewards were taken relative to continuing to give all patients the current treatment. Thus if this treatment is recommended, the gain to patients outside the trial is taken to be zero, that is $g_{2}=0$. The gain per patient outside the trial if the experimental treatment is recommended was taken to be of the form $g_{1}\left(\xi_{1}\right)\;=\;l\left(1\;-\;\Phi \left(z_{\alpha /2}\;-\;\left(z_{\alpha /2}\;+\;z_{\beta }\right)\theta /\theta_{1}\right)\right)\;-\;m$ where $z_{\alpha }\;=\;\Phi^{-1}\left(1\;-\;\alpha \right)$, $\theta \;=\;\text{logit}\left(\xi_{1}\right)\;-\;\text{logit}\left(\xi_{0}\right)$, $\theta_{1}=\text{logit}\left(\xi_{0}+\delta_{0}\right)-\text{logit}\left(\xi_{0}\right)$, and logit(ξ)=log(ξ/(1−ξ)) for some *l*, α, β, and δ_0_. This form reflects a fixed cost of *m* per patient with a gain of *l* per patient if the treatment is shown to be effective in the subsequent trial where that trial has frequentist (one‐sided) type I error rate of α/2 and power 1−β to detect a log‐odds ratio of θ_1_. Stallard assumed linear discounting for *j* less than some constant, *n*_0_, with geometric discounting for $j>n_{0}$ whereas we will assume geometric discounting for all *j*. Patients in the trial were taken to have constant (discounted) cost, that is $h_{1}\left(\xi_{1}\right)=-k$, for some *k*. Since we know $n_{2}=0$, it is not necessary to specify *h*_2_. The parameter ξ_1_ was again taken to have a Beta(*a*_1_, *b*_1_) prior distribution. As the gain function $g_{2}\left(\xi_{2}\right)$ does not depend on ξ_2_, it is not necessary to specify a value for the point‐prior for this parameter in this case.

Since $g_{2}\left(\xi_{2}\right)=0$ for all ξ_2_, the expression (12) becomes

(15) $$n_{1}^{*}=\sqrt{\frac{-Ng_{1}^{\prime }\left(g_{1}^{-1}\left(0\right)\right)v_{1}\left(g_{1}^{-1}\left(0\right)\right)\pi \left(g_{1}^{-1}\left(0\right)\right)}{2(E_{0}\left(h_{1}\left(\xi_{1}\right)\right)-E_{0}\left(\max\left\{g_{1}\left(\xi_{1}\right),0\right\}\right))}}$$

where $\pi (\xi_{1})$ is the (univariate) prior density for ξ_1_. The value of $E_{0}\left(\max\left\{g_{1}\left(\xi_{1}\right),0\right\}\right)$ can be evaluated using numerical integration and, as $h_{1}=-k$, we have $E_{0}\left(h_{1}\left(\xi_{1}\right)\right)=-k$.

**Figure 3:**
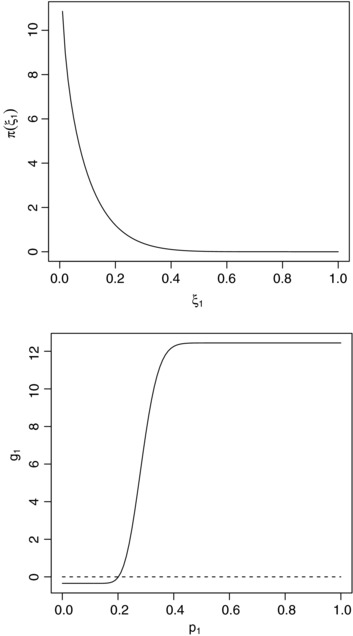
Prior distribution for ξ_1_ (upper panel) and gain function $g_{1}\left(\xi_{1}\right)$ giving gain from treating each future patient with treatment 1 (lower panel). The gain function $g_{2}\left(\xi_{2}\right)=0$ is shown as a dashed line on the right hand panel for comparison (see text for details).

**Figure 4:**
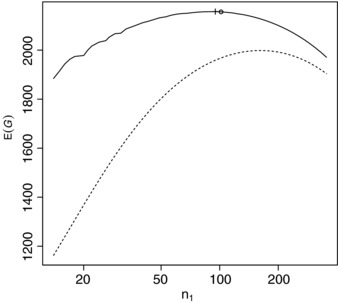
Prior expected gain (solid line) and approximate prior expected gain (dashed line) for a range of *n*_1_ for the second single‐arm Bernoulli example with N=5000 (see text for details of gain function and prior distribution parameter values). The optimal and approximately optimal values of *n*_1_ are marked by + and ○ respectively

Although the form of utility function proposed by Stallard (1998) was not exactly that proposed here, based on values given, we took λ=exp(−0.00173)=0.99827, l=12.79, m=0.346, α=0.05, β=0.1, $\xi_{0}=0.2$, $\delta_{0}=0.15$, $a_{1}=0.845$ and $b_{1}=9.155$. Figure 3 shows the prior distribution for ξ_1_ along with the form of $g_{1}\left(\xi_{1}\right)$ in this case. Figure 4 shows the prior expected gain calculated exactly using the betabinomal prior predictive distribution for $\sum_{j=1}^{n_{1}}Y_{1j}$ for a range of values of *n*_1_ values, plotted on a logarithmic scale, along with the approximation given by (7). It can be seen that in this case the approximation (7) to the expected gain is rather poor, particularly for smaller *n*_1_. The value of $n_{1}^{*}$ obtained using (15) in this case is 102. This value and the associated prior expected gain is marked on the figure by a circle. Note again that $n_{1}^{*}$ does not maximise the approximate gain given by (7) that is shown by the dashed line on the plot. In this case *n*_1_ the value of $n_{1}^{*}$ is quite far from the value of *n*_1_ maximizing the approximate gain, though it is closer to the true optimal value of 95, which marked on the figure by a plus sign.

**Figure 5:**
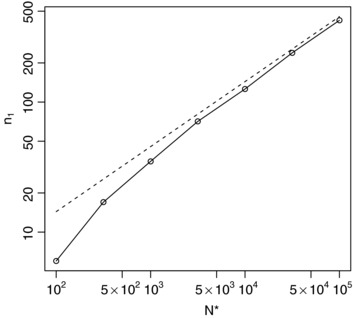
Optimal (solid lines with points) and approximately optimal (dashed lines) values for *n*_1_ for a range of *N* values for the second single‐arm Bernoulli example (see text for details of gain function and prior distribution parameter values).

Figure 5 gives values of $n_{1}^{*}$ from (15) along with exact optima for a range of values of *N*, again with both plotted on a logarithmic scale. The approximation given by (15) is again close to the true value, and approaches it as *N* increases as would be expected.

The effect of varying the prior distribution for ξ_1_ was again investigated, and again illustrated in Web Appendix B. Web Figure 2 shows the optimal sample size $n_{1}^{*}$ with N=5000 for a range of $n_{01}=a_{1}+b_{1}$ and $y_{01}=a_{1}/\left(a_{1}+b_{1}\right)$ values. In this case as *y*_01_ increases from 0.04 to 0.0845 the optimal sample size is increased in a similar way to that noted for the example above. In this case as *y*_01_ increases further, however, the optimal sample size is reduced. Here, since $h_{i}=-k$, there is a cost associated with experimentation so that when there is strong prior belief that treatment 1 is superior to treatment 2, rather than giving many patients treatment 1 in a trial, a smaller trial is optimal.

At the end of the trial, treatment 1 will be recommended if $E_{\xi |Y}\left(g_{1}\left(\xi_{1}\right)|Y\right)>0$. For large *n*_1_, that is approximately if $E_{\xi |Y}\left(\xi_{1}|Y\right)>g_{1}^{-1}\left(0\right)$, which, using expression (1), is true when $n_{1}{\bar{Y}}_{1}>g_{1}^{-1}\left(0\right)\left(n_{01}+n_{1}\right)-n_{01}y_{01}$. Considering recommendation of treatment 1 to correspond to rejection of the null hypothesis that $\xi_{1}=g_{1}^{-1}\left(0\right)=0.201$, frequentist error rates attained for the optimal designs obtained can be derived.

With a prior distribution with $a_{1}=0.845$ and $b_{1}=9.155$, taking $n_{1}=102$ gives a type I error rate of 0.39. The form of $g_{1}\left(\xi_{1}\right)$ shown in Fig. 3 suggests that we might require a test with high power when ξ=0.35, since this value of ξ_1_ is associated with a high gain value. The power of the optimal test in this case is 0.999.

Type I and type II error rate values for the optimal designs and prior distributions considered in Web Fig. 2 are shown in Web Fig. 3. As the prior weight becomes small, the optimal decision at the end of the trial is to select treatment 1 whenever it has observed mean exceeding $g_{1}^{-1}\left(0\right)$, so the type I error rate approaches 0.5. As the prior weight increases, since in this case the optimal value of *n*_1_ is relatively small, prior information comes to dominate the final decision and the type I error rate approaches zero or one, with the type II error approaching one or zero, depending on whether the prior mean is less than or greater than $g_{1}^{-1}\left(0\right)$.

### 4.2 A two‐arm trial with Poisson data

The third example is based on an example given by Berry et al. (1994), who describe a trial of an HIB vaccine in Navajo children aged 2–18 months. The number of HIB cases is assumed to follow a Poisson distribution. Rather than expressing *n*_1_, *n*_2_, and *N* in terms of child‐months, we will assume that all children are followed up for the entire 16‐month period, and refer to the number of children in the trial and population. The observed number of cases per child *j* in group *i*, will be denoted by $Y_{ij},j=1,\ldots ,n_{i},i=1,2$. The distribution of $Y_{ij}$ can be parameterised such that ξ_1_ and ξ_2_ are the expected numbers of cases per child for treatments 1 (the new vaccine) and 2 (the placebo), respectively. Thus $p\left(y_{ij}|\xi_{i}\right)=\xi_{i}^{y_{ij}}\exp\left(-\xi_{i}\right)/y_{ij}!,j=1,\ldots ,n_{i},i=1,2$, so that $y_{ij}$ has mean $\xi_{i}$, with $\xi_{i}$ following independent prior gamma($\alpha_{i}$, $\beta_{i}$) distributions, that is with density $\pi \left(\xi_{1},\xi_{2}\right)=\prod_{i=1}^{2}\xi_{i}^{\alpha_{i}-1}\exp\left(-\xi_{i}\beta_{i}\right)\beta_{i}^{\alpha_{i}}/\Gamma \left(\alpha_{i}\right)$ for some $\alpha_{i},\beta_{i},i=1,2$. Note that $\xi_{i}$ has prior mean $\alpha_{i}/\beta_{i}$ and prior variance $\alpha_{i}/\beta_{i}^{2}$. The posterior distribution of $\xi_{i}$ given Y is a gamma ($\alpha_{i}+\sum_{j=1}^{n_{i}}y_{ij}$, $\beta_{i}+n_{i}$) distribution and the prior predictive distribution of $\sum_{j=1}^{n_{i}}y_{ij}$ is NegBin ($\alpha_{i},{\left(1+n_{i}/\beta_{i}\right)}^{-1}$).

Berry et al. (1994) include in their gain function a term that depends on the observed data that reflects the probability of obtaining regulatory approval for the vaccine. Here, we assume the gain from a child receiving treatment *i* depends on $\xi_{i}$ alone and, since $\xi_{i}$ gives the rate of HIB cases, which we would like to minimize, we take gain functions $h_{i}\left(\xi_{i}\right)=g_{i}\left(\xi_{i}\right)=-\xi_{i}$, i=1,2. The case of gain functions that depend on the observed data is considered briefly in the discussion section below.

The optimal values may be approximated using expressions (10) and (11). In this case $v_{i}\left(\xi_{i}\right)=\xi_{i}$, $g_{i}^{\prime }\left(\xi_{i}\right)=-1$, and $g_{2}^{-1}\left(g_{1}\left(\xi \right)\right)=g_{1}^{-1}\left(g_{2}\left(\xi \right)\right)=\xi$ so that, for example, $n_{1}^{*}$ is

$$\begin{array}{c} \sqrt{\frac{N\int \xi \pi (\xi ,\xi )d\xi }{2(E_{0}\left(\max_{i=1,2}\left(-\xi_{i}\right)\right)-E_{0}\left(-\xi_{1}\right))}}=\sqrt{\frac{N\int \xi \pi (\xi ,\xi )d\xi }{2(E_{0}\left(\xi_{1}\right)-E_{0}\left(\min_{i=1,2}\xi_{i}\right))}}, \end{array}$$

the integral in the numerator being equal to

$$\begin{array}{c} \int \xi \frac{\xi^{\alpha_{1}-1}\exp\left(-\xi \beta_{1}\right)\beta_{1}^{\alpha_{1}}\xi^{\alpha_{2}-1}\exp\left(-\xi \beta_{2}\right)\beta_{2}^{\alpha_{2}}}{\Gamma \left(\alpha_{1}\right)\Gamma \left(\alpha_{2}\right)}d\xi =\frac{\Gamma \left(\alpha_{1}+\alpha_{2}\right)\beta_{1}^{\alpha_{1}}\beta_{2}^{\alpha_{2}}}{\Gamma \left(\alpha_{1}\right)\Gamma \left(\alpha_{2}\right){\left(\beta_{1}+\beta_{2}\right)}^{\left(\alpha_{1}+\alpha_{2}\right)}}. \end{array}$$

**Figure 6:**
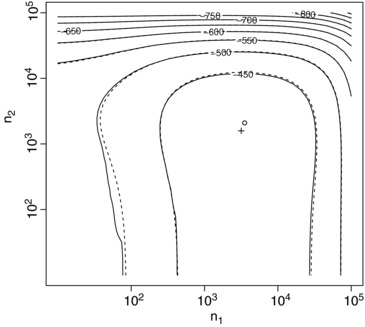
Contour plot of prior expected gain (solid lines) and approximate prior expected gain (dashed lines) for N=108,000 for a range of *n*_1_ and *n*_2_ values assuming gamma (1, 200) and gamma (5, 667) prior distributions. The optimal and approximately optimal values of *n*_1_ and *n*_2_ are marked by + and ○, respectively.

Following Berry et al. (1994), we take $\left(\alpha_{1},\beta_{1}\right)=\left(1,200\right)$ and $\left(\alpha_{2},\beta_{2}\right)=\left(5,667\right)$, the latter corresponding to the placebo (note that the $\beta_{i}$ values given by Berry et al. are 16 times those used here as they take $\xi_{i}$ to give the rate of cases per child‐month). Berry et al. report that approximately 5400 Navajo are born each year so that minimization of HIB cases over a 20‐year period would correspond to N=108,000. Figure 6 shows a contour plot giving the prior expected gain for this *N* for a range of *n*_1_ and *n*_2_ values (plotted on logarithmic scales) together with the approximation given by (7) (dashed lines). It can be seen that even for small sample sizes, (7) gives a close approximation to the true prior expected gain. The optimal design has $n_{1}=3162$ and $n_{2}=1585$, and is marked by the plus sign. The approximately optimal design given by (10) and (11) has $n_{1}^{*}=3524$ and $n_{2}^{*}=2089$, and is marked by a circle. The prior expected gain, in this case corresponding to minus one times the prior expected number of HIB cases in the population over the 20 year period, is −416.9 using the optimal design and −417.4 using the approximately optimal design.

**Figure 7:**
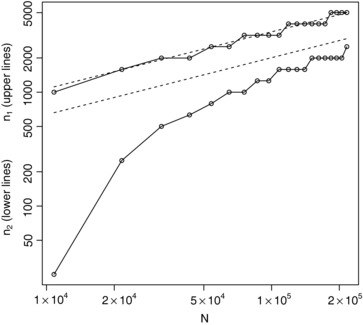
Optimal (solid lines with points) and approximately optimal (dashed lines) values for *n*_1_ (upper lines) and *n*_2_ (lower lines) for a range of *N* values assuming gamma (1, 200) and gamma (5, 667) prior distributions.

Figure 7 shows the values of $n_{1}^{*}$ and $n_{2}^{*}$ along with the approximations from (10) and (11) (dashed lines) for a range of *N* values, again with both plotted on logarithmic scales. It can again be seen how the approximations become increasingly accurate as *N* increases.

The effect of varying the prior distributions for ξ_1_ and ξ_2_ was again investigated. Web Fig. 4 in Web Appendix B shows the optimal sample sizes, $n_{1}^{*}$ and $n_{2}^{*}$, for a range of prior means and prior weights, here equal for the two priors, when N=108,000. When the prior means are equal, the optimal sample size increases with prior weight. For unequal prior means, more patients are assigned to the arm considered *a priori* to be superior, in this case corresponding to a lower prior mean since $h_{i}\left(\xi_{i}\right)=g_{i}\left(\xi_{i}\right)=-\xi_{i}$, with the number assigned to the inferior arm increasing with prior weight when this is small and decreasing for larger prior weight values. Web Fig. 5 shows the effect of changing the prior weight for ξ_1_ alone when the prior mean is equal to, greater than or less than that for ξ_2_. In this case increasing the prior weight leads to an increase in the optimal sample size for both arms, with the arm with the lower prior mean having a smaller optimal sample size.

The examples above compare the large sample approximation for the optimal sample size for arm *i*, $n_{i}^{*}$, given by expression (10), with that obtained by exact numerical optimization in two examples, enabling assessment of the approximation for smaller values of *N*. The derivation of (10) relies on large sample approximations in two ways; first the distribution of the posterior expected utility $E(g_{i}\left(\xi_{i}\right)|\bar{Y})$ is approximated by its asymptotic normal form in (5) using the central limit theorem and the delta method, and second, the derivative of the expected gain, given by (8) is approximated by (9). The first approximation is exact when $Y_{ij}$ are normally distributed and $g_{i}\left(\xi_{i}\right)$ are linear. The first and second examples suggest that both approximations are sufficiently accurate for Bernoulli data even for quite small *N* when $g_{i}\left(\xi_{i}\right)$ are linear, but less so for nonlinear $g_{i}\left(\xi_{i}\right)$, when the first approximation may be poor, as noted by Bernado and Smith (2000). For nonnormal data the accuracy of the first approximation also improves as the prior weight $n_{0i}$ increases, though, as illustrated in the third example above, this also leads to smaller $n_{i}^{*}$, so that the overall accuracy of the asymptotic approximation to the optimal sample size might be poorer.

## 5 Discussion

The work reported above leads to expressions for the optimal sample size in a clinical trial to compare two treatments or to compare a single experimental treatment with a historical control the properties of which are assumed known. The observed data for patients receiving treatment *i* are assumed to follow a distribution of one parameter exponential family form, with mean $\xi_{i}$ assumed to have a conjugate prior distribution. Optimization is based on consideration of the costs and benefits both from patients in the trial, given by some utility function, $h_{i}\left(\xi_{i}\right)$ for patients receiving treatment *i*, and from subsequent patients who will receive treatment *i* based on the results of the trial, given by some function $g_{i}\left(\xi_{i}\right)$ if they receive treatment *i*. Although the expressions obtained could be directly used to design a trial, it is also of more general interest to see how the optimal sample size depends on the size of the population under investigation. We have shown that if the population is assumed to be of some known size, *N*, for any $h_{i}$ and $g_{i}$ satisfying sufficient regularity conditions for expectations to exist, differentiable with $g_{i}$ strictly increasing, and satisfying the condition given by (2); that is $E_{0}\left(h_{i}\left(\xi_{i}\right)\right)\leq E_{0}\left(\max_{i=1,2}\left(g_{i}\left(\xi_{i}\right)\right)\right)$, the optimal sample size is $O(N^{1/2})$ as N→∞. If it is assumed that there is an infinite population with geometric discounting with discounting factor λ, under the same conditions the optimal sample size is $O(N^{*1/2})$ as $N^{*}\to \infty$ where $N^{*}={\left(1-\lambda \right)}^{-1}$. This extends previous work by Cheng et al. (2003).

Although we have considered general functions $h_{i}\left(\xi_{i}\right)$ and $g_{i}\left(\xi_{i}\right)$, giving the gain to patients inside and outside the trial who receive treatment *i*, i=1,2, we have assumed that these are functions of $\xi_{i}$ only. This is a common assumption and it seems reasonable that the benefit to a patient from taking a given treatment will depend only on the properties of that treatment (see, e.g. Lindley, 1997, who cites the seminal work by Raiffa and Schlaifer, 1961). Noting, however, that the gain functions $g_{i}$ correspond to gain from future patients if the trial indicates that treatment *i* is superior, some authors have proposed gain functions for patients treated following the trial that depend, in addition to $\xi_{i}$, on the observed trial data, Y. In particular, gain functions have been proposed that reflect the fact that use of a novel treatment following a trial may depend on regulatory decisions that in turn depends on whether trial results are sufficiently compelling (see, e.g. Posch and Bauer, 2013). In both of the more realistic examples described above, the gain functions given by Stallard (1998) and Berry et al. (1994) depended on Y, and we have simplified the gain functions when discussing these examples above.

The forms of the utility functions $h_{i}$ and $g_{i},i=1,2$ given above were motivated by consideration of the gain to each patient from participation in the trial or from being treated with treatment *i* following the trial, suggesting that the trial sample size is optimised from the patient's perspective. The general form of the expected gain given by (3), however, can express any gain so long as this can be specified on a per‐patient basis. The results obtained could thus also apply to financial gains from a commercial perspective or to societal gains from development of a novel therapy. In the latter cases it may be more appropriate for $h_{i}$ and $g_{i}$ to have a more complex form or to depend on trial data as discussed above.

The condition (2) ensures that the gain per patient in the trial does not exceed that per patient outside the trial if patients were to receive optimal treatment. If this does not hold the optimal design will be to continue with trial forever, giving all patients the treatment for which the prior expected gain $E_{0}\left(h_{i}\left(\xi_{i}\right)\right)$ is the largest. This restriction on $h_{i}$ and $g_{i}$ seems reasonable if $h_{i}$ reflects not only the benefit to patients in the trial receiving treatment *i*, but also the cost of the trial, either in financial terms for the trial sponsor or funder or in terms of commitment by the patient, both of which may be considerable.

It is interesting to compare the optimal sample sizes obtained above with sample sizes typical for clinical trials. In particular, it might be of interest to consider the size of population for which conventional sample sizes would correspond to that of the optimal design. A method for frequentist sample size calculations for a single arm trial with a Bernoulli response is given by Fleming (1982), who shows that the sample size required for a trial with (one‐sided) type I error rate α and power 1−β to detect an improvement to a success probability of *p*_1_ from a control success probability of *p*_0_ as $p_{1}\to p_{0}$ is ${\left(\sqrt{p_{0}\left(1-p_{0}\right)}\Phi^{-1}\left(1-\alpha \right)+\sqrt{p_{1}\left(1-p_{1}\right)}\Phi^{-1}\left(1-\beta \right)\right)}^{2}/{\left(p_{1}-p_{0}\right)}^{2}$. As discussed above, the form of $g_{1}\left(\xi_{1}\right)$ given in the second single arm Bernoulli data example above and shown in Fig. 3 suggests that an appropriate value for *p*_1_ might be about 0.35, since this value of ξ_1_ is associated with a high gain value. For α=0.025 and β=0.9, this would give a sample size of 111. Using the gain function described above, this would be optimal for a population of size, or expected population size in the case of geometric discounting, of about 3000. The prior distribution used in the example above, and also shown in Figure 3 is such that a value of ξ_1_ as large as 0.35 is highly unlikely, suggesting that a smaller value could be used for *p*_1_ in the frequentist sample size calculation. The 95th percentile of the prior distribution is 0.256. To give power of 0.9 to detect a treatment effect corresponding to this value of *p*_1_ would require a sample size of 704. This would be optimal for an expected population of size of a little over 100,000. It is important to note that even when the sample sizes are similar, the optimal designs obtained above may be very different from those obtained using the usual frequentist approach as, following the trial, a treatment is recommended depending on the posterior expected gains rather than on the basis of type I error rate control. As seen above, depending on the prior distribution, this can lead to type I error rates considerably higher than those conventionally used in large‐scale confirmatory studies. In this regard, the designs obtained are more similar to those sometimes used for early‐phase clinical trials or pilot studies (Schoenfeld, 1980; Stallard, 2012). Further comparison of frequentist and decision‐theoretic approaches is an area where further research would be of interest.

## Conflict of interest

*The authors have declared no conflict of interest*.

## Supporting information

Stallard et al Program 1

Stallard et al Program 2

**Web Figure 1**: Optimal sample size for first single arm Bernoulli example with $\xi_{2}=0.5,h_{i}\left(\xi_{i}\right)=g_{i}\left(\xi_{i}\right)=\xi_{i}$ and *N* = 100 for varying weights, *n*_01_ for the prior distribution for ξ_1_ when the prior mean is 0.3 (left hand panel), 0.5 (centre panel) or 0.7 (right hand panel).
**Web Figure 2**: Optimal sample size for second single arm Bernoulli example with $h_{1}\left(\xi_{1}\right)=-k,g_{2}\left(\xi_{2}\right)=0$ and $g_{1}\left(\xi_{1}\right)=l(1-\Phi \left(z_{\alpha /2}-\left(z_{\alpha /2}+z_{\beta }\right)\theta /\theta_{1}\right)-m$ as described in the main text and *N* = 5000 for varying weights, *n*_01_ for the prior distribution for ξ_1_ when the prior mean is 0.04 (left hand panel), 0.0845 (centre panel) or 0.4 (right hand panel).
**Web Figure 3**: Type I (dashed line) and type II (dotted line) error rates for the optimal designs shown in Web Figure 2.
**Web Figure 4**: Optimal sample sizes for treatment group 1 (solid line) and treatment group 2 (dashed line) for two arm Poisson example with $h_{i}\left(\xi_{i}\right)=g_{i}\left(\xi_{i}\right)=-\xi_{i}$ and *N* = 10800 for varying weights, $n_{01}=n_{02}$, when the prior mean for treatment group 1 is 0.05 and the prior mean for treatment group 2 is 0.05 (left panel), 0.075 (centre panel) or 0.1 (right panel).
**Web Figure 5**: Optimal sample sizes for treatment group 1 (solid line) and treatment group 2 (dashed line) for two arm Poisson example with $h_{i}\left(\xi_{i}\right)=g_{i}\left(\xi_{i}\right)=-\xi_{i}$ and *N* = 10800 for varying weights *n*_01_, with *n*_02_ = 10, when the prior means for treatment groups 1 and 2 are respectively 0.1 and 0.05 (left panel), 0.05 and 0.05 (centre panel) or 0.05 and 0.1 (right panel).
