# Supplementary material for: Determination of the optimal sample size for a clinical trial accounting for the population size
Source: Biom J. 2016 May 17;59(4):609–25. doi: 10.1002/bimj.201500228 (PMC5516263; doi:10.1002/bimj.201500228)
Supplement: Supplementary file 3 — Web Figure 1: Optimal sample size for first single arm Bernoulli example with ξ2=0.5,hi(ξi)=gi(ξi)=ξi and N = 100 for varying weights, n 01 for the prior distribution for ξ1 when the prior mean is 0.3 (left hand panel), 0.5 (centre panel) or 0.7 (right hand panel). Web Figure 2: Optimal sample size for second single arm Bernoulli example with h1(ξ1)=−k,g2(ξ2)=0 and g1(ξ1)=l(1−Φ(zα/2−(zα/2+zβ)θ/θ1)−m as described in the main text and N = 5000 for varying weights, n 01 for the prior distribution for ξ1 when the prior mean is 0.04 (left hand panel), 0.0845 (centre panel) or 0.4 (right hand panel). Web Figure 3: Type I (dashed line) and type II (dotted line) error rates for the optimal designs shown in Web Figure 2. Web Figure 4: Optimal sample sizes for treatment group 1 (solid line) and treatment group 2 (dashed line) for two arm Poisson example with hi(ξi)=gi(ξi)=−ξi and N = 10800 for varying weights, n01=n02, when the prior mean for treatment group 1 is 0.05 and the prior mean for treatment group 2 is 0.05 (left panel), 0.075 (centre panel) or 0.1 (right panel). Web Figure 5: Optimal sample sizes for treatment group 1 (solid line) and treatment group 2 (dashed line) for two arm Poisson example with hi(ξi)=gi(ξi)=−ξi and N = 10800 for varying weights n 01, with n 02 = 10, when the prior means for treatment groups 1 and 2 are respectively 0.1 and 0.05 (left panel), 0.05 and 0.05 (centre panel) or 0.05 and 0.1 (right panel). [file BIMJ-59-609-s003.pdf]

Web-based Supplementary Materials for

Determination of the optimal sample size for a clinical trial accounting for the population size

Nigel Stallard, Frank Miller, Simon Day, Siew Wan Hee, Jason Madan, Sarah Zohar and Martin Posch

Web Appendix A: Derivation of derivative of expected utility and its large sample approximation

We wish to find a large-sample approximation to the prior expected value of the derivative

$$\frac{\partial \mathcal{G}}{\partial n_1} = h_1(\xi_1) - E_{\mathbf{Y}}(\max_{i=1,2} E_{\xi|\mathbf{Y}}(g_i(\xi_i) \mid \mathbf{Y})) + (N - n_1 - n_2)\frac{\partial E_{\mathbf{Y}}(\max_{i=1,2} E_{\xi|\mathbf{Y}}(g_i(\xi_i) \mid \mathbf{Y}))}{\partial n_1}. \quad (1)$$

Equation (6) in the main text states that

$$E_{\mathbf{Y}}(\max_{i=1,2} E_{\xi|\mathbf{Y}}(g_i(\xi_i) \mid \mathbf{Y})) \to \tilde{\xi}_1 \Phi(\delta) + \tilde{\xi}_2 \Phi(-\delta) + a\phi(\delta) \quad (2)$$

where

$$\tilde{\xi}_i = g_i\left((n_{0i}y_{0i} + n_i\xi_i)/(n_{0i} + n_i)\right),$$

$$a^2 = \sum_{i=1}^{2} n_i \left(g_i'(\xi_i)\right)^2 v_i(\xi_i)/(n_{0i} + n_i)^2,$$

$$\delta = (\tilde{\xi}_1 - \tilde{\xi}_2)/a$$

and ϕ and Φ denote standard normal density and distribution functions.

Differentiating the right hand side of (2) with respect to n_1, noting that $d\Phi(x)/dx = \phi(x)$ and $d\phi(x)/dx = -x\phi(x)$, gives

$$\Phi(\delta)\frac{\partial \tilde{\xi}_1}{\partial n_1} + \tilde{\xi}_1 \phi(\delta)\frac{\partial \delta}{\partial n_1} - \tilde{\xi}_2 \phi(\delta)\frac{\partial \delta}{\partial n_1} - a\delta\phi(\delta)\frac{\partial \delta}{\partial n_1} + \phi(\delta)\frac{\partial a}{\partial n_1},$$

which is equal to

$$\Phi(\delta)\frac{\partial \tilde{\xi}_1}{\partial n_1} + (\tilde{\xi}_1 - \tilde{\xi}_2 - a\delta)\phi(\delta)\frac{\partial \delta}{\partial n_1} + \phi(\delta)\frac{\partial a}{\partial n_1},$$

which, since $a\delta = \tilde{\xi}_1 - \tilde{\xi}_2$, is equal to

$$\Phi(\delta)\frac{\partial \tilde{\xi}_1}{\partial n_1} + \phi(\delta)\frac{\partial a}{\partial n_1}.$$

Differentiation yields

$$\frac{\partial \tilde{\xi}_1}{\partial n_1} = \frac{g_1'(\xi_1)n_{01}(\xi_1 - y_{01})}{(n_{01}+n_1)^2}$$

and

$$\frac{\partial a}{\partial n_1} = \frac{(n_{01}-n_1)v_1(\xi_1)\left(g_1'(\xi_1)\right)^2}{2a(n_{01}+n_1)^3}$$

so that the derivative of the right hand side of (2) is equal to

$$\Phi(\delta)\frac{g_1'(\xi_1)n_{01}(\xi_1 - y_{01})}{(n_{01}+n_1)^2} + \phi(\delta)\frac{(n_{01}-n_1)v_1(\xi_1)\left(g_1'(\xi_1)\right)^2}{2a(n_{01}+n_1)^3}.$$

As both $n_1 \to \infty$ and $n_{02}+n_2 \to \infty$, we have $a \to 0$ and

$$\Phi(\delta)\frac{\partial \tilde{\xi}_1}{\partial n_1} \to (I(\delta \geq 0) + I(\delta > 0))n_{01}g_1'(\xi_1)(\xi_1 - y_{01})/(2n_1^2)$$

where I denotes an indicator function taking the value 1 if its argument is true and 0 otherwise. Since $E_0(\xi_1) = y_{01}$ and $g_1'(\xi_1)$ and n_{01} are assumed to be finite, we thus have

$$E_0\left(\Phi(\delta)\frac{\partial \tilde{\xi}_1}{\partial n_1}\right) = o(n_1^{-2}).$$

Again as both n_1 and $n_{02}+n_2$ become large, the term $(n_{01}-n_1)/(n_{01}+n_1)^3$ in $\partial a/\partial n_1$ approaches n_1^{-2}, so that, since $a \to 0$, writing $E_0(\phi(\delta)\partial a/\partial n_1)$ as an integral over the prior density for ξ_1 and ξ_2, which will be denoted $\pi(\xi_1,\xi_2)$ gives

$$E_0\left(\phi(\delta)\frac{\partial a}{\partial n_1}\right) \to \frac{1}{n_1^2}\lim_{a\to 0}\int -\frac{1}{a}\phi\left(\frac{\tilde{\xi}_1-\tilde{\xi}_2}{a}\right)g_1'(\xi_1)\frac{v_1(\xi_1)}{2}\left(g_1'(\xi_1)\right)\pi(\xi_1,\xi_2)d\xi_1 d\xi_2.$$

As $a \to 0$, the term

$$\frac{1}{a}\phi\left(\frac{\tilde{\xi}_1-\tilde{\xi}_2}{a}\right)$$

in the integrand approaches a Dirac-δ function that is non-zero only when $\tilde{\xi}_1 - \tilde{\xi}_2 = 0$, integrates to one. For n_{02} finite, so that n_1 and n_2 are both infinite, $\tilde{\xi}_i \to g_i(\xi_i)$ so that $\tilde{\xi}_1 - \tilde{\xi}_2 = 0$ when $\xi_1 = g_1^{-1}(g_2(\xi_2))$. For n_{02} infinite, if n_2 is finite, we have $\tilde{\xi}_2 = g_2(y_{02})$, so that $\tilde{\xi}_1 - \tilde{\xi}_2 = 0$ when $\xi_1 = g_1^{-1}(g_2(y_{02}))$, which, since in this case the prior distribution for xi_2 has mass only at y_{02} can again be written as $\xi_1 = g_1^{-1}(g_2(\xi_2))$. We thus have

$$E_0\left(\phi(\delta)\frac{\partial a}{\partial n_1}\right) \to \frac{1}{n_1^2}\int -\frac{v_1(g_1^{-1}(g_2(\xi_2)))}{2}\left(g_1'(g_1^{-1}(g_2(\xi_2)))\right)\pi(g_1^{-1}(g_2(\xi_2)),\xi_2)d\xi_2,$$

and hence

$$E_0\left(\phi(\delta)\frac{\partial a}{\partial n_1}\right) = O(n_1^{-2})$$

This is thus the dominant term in $E_0\left(\partial E_{\mathbf{Y}}(\max_{i=1,2} E_{\xi|\mathbf{Y}}(g_i(\xi_i) \mid \mathbf{Y}))/\partial n_1\right)$ so that

$$E_0\left(\frac{\partial E_{\mathbf{Y}}(\max_{i=1,2} E_{\xi|\mathbf{Y}}(g_i(\xi_i) \mid \mathbf{Y}))}{\partial n_1}\right) \to \left(\frac{1}{n_1^2}\int -\frac{v_1(g_1^{-1}(g_2(\xi_2)))}{2}\left(g_1'(g_1^{-1}(g_2(\xi_2)))\right)\pi(g_1^{-1}(g_2(\xi_2)),\xi_2)d\xi_2\right).$$

Substituting into (1), noting that as n_1 and $n_{02}+n_2$ become large we have $E_{\mathbf{Y}}(\max_{i=1,2} E_{\xi|\mathbf{Y}}(g_i(\xi_i) \mid \mathbf{Y})) \to \max_{i=1,2} g_i(\xi_i)$, for large n_1 and $n_{02}+n_2$ we thus get

$$E_0(\partial\mathcal{G}/\partial n_1) \to E_0(h_1(\xi_1) - \max_{i=1,2} g_i(\xi_i)) + \frac{N-n_1-n_2}{n_1^2}\int -\frac{v_1(g_1^{-1}(g_2(\xi_2)))}{2}\left(g_1'(g_1^{-1}(g_2(\xi_2)))\right)\pi(g_1^{-1}(g_2(\xi_2)),\xi_2)d\xi_2$$

which, since N will dominate n_1 and n_2 as $N\to\infty$, tends to

$$E_0(h_1(\xi_1) - \max_{i=1,2} g_i(\xi_i)) + \frac{N}{n_1^2}\int -\frac{v_1(g_1^{-1}(g_2(\xi_2)))}{2}\left(g_1'(g_1^{-1}(g_2(\xi_2)))\right)\pi(g_1^{-1}(g_2(\xi_2)),\xi_2)d\xi_2.$$

Setting this derivative to zero and solving for n_1 gives

$$n_1^* = \sqrt{\frac{N\int v_1(g_1^{-1}(g_2(\xi_2)))g_1'(g_1^{-1}(g_2(\xi_2)))\pi(g_1^{-1}(g_2(\xi_2)),\xi_2)d\xi_2}{2(E_0(\max_{i=1,2} g_i(\xi_i)) - E_0(h_1(\xi_1)))}}$$

as stated in the main text.

Web Appendix B: Investigation of effect of varying the prior distribution in the examples

Figures 1-4 illustrate the effect of varying the prior distribution in the three examples in the paper (see main text for details and discussion of the results obtained).

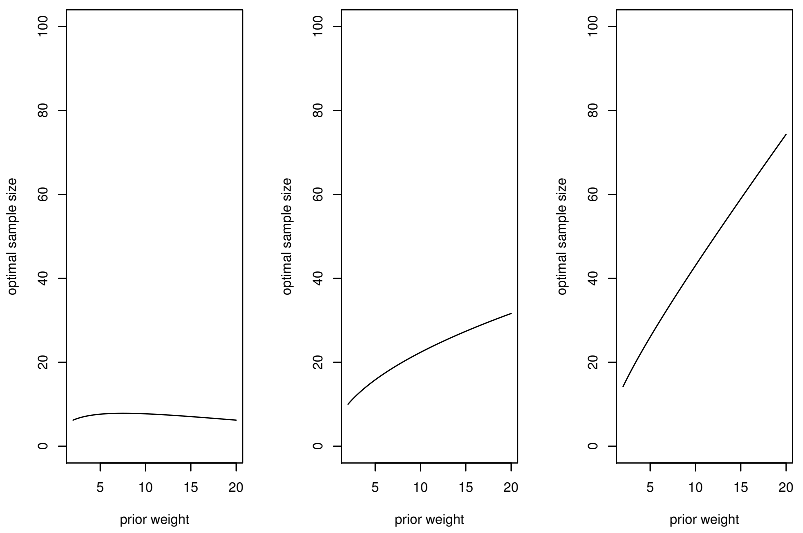

Web Figure 1: Optimal sample size for first single arm Bernoulli example with $\xi_2 = 0.5$, $h_i(\xi_i) = g_i(\xi_i) = \xi_i$ and $N = 100$ for varying weights, n_{01} for the prior distribution for ξ_1 when the prior mean is 0.3 (left hand panel), 0.5 (centre panel) or 0.7 (right hand panel).

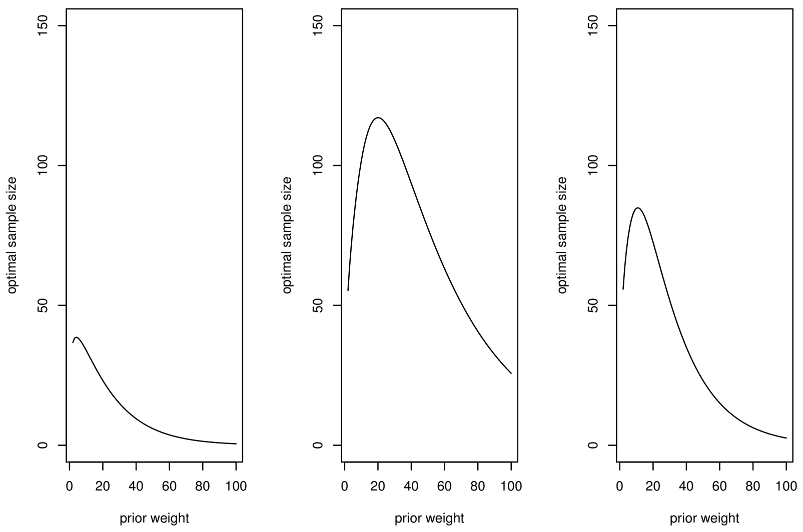

Web Figure 2: Optimal sample size for second single arm Bernoulli example with $h_1(\xi_1) = -k$, $g_2(\xi_2) = 0$ and $g_1(\xi_1) = l(1 - \Phi(z_{\alpha/2} - (z_{\alpha/2} + z_\beta)\theta/\theta_1) - m$ as described in the main text and $N = 5000$ for varying weights, n_{01} for the prior distribution for ξ_1 when the prior mean is 0.04 (left hand panel), 0.0845 (centre panel) or 0.4 (right hand panel).

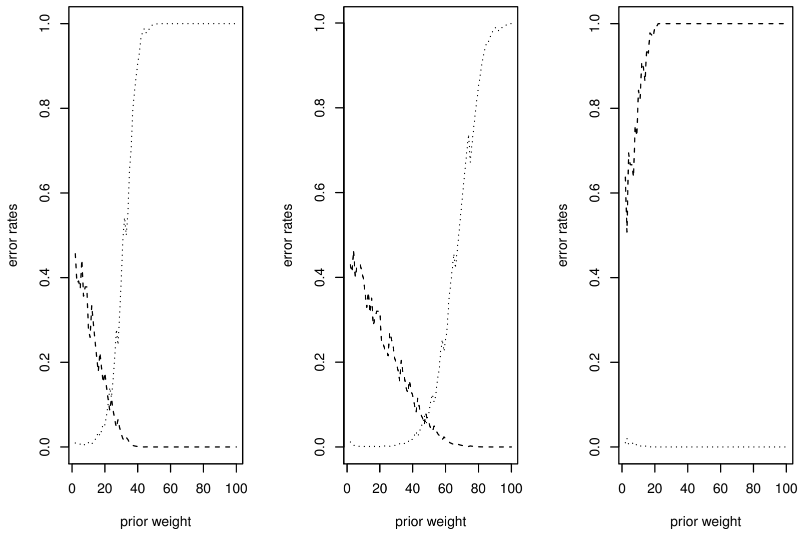

Web Figure 3: Type I (dashed line) and type II (dotted line) error rates for the optimal designs shown in Web Figure 2.

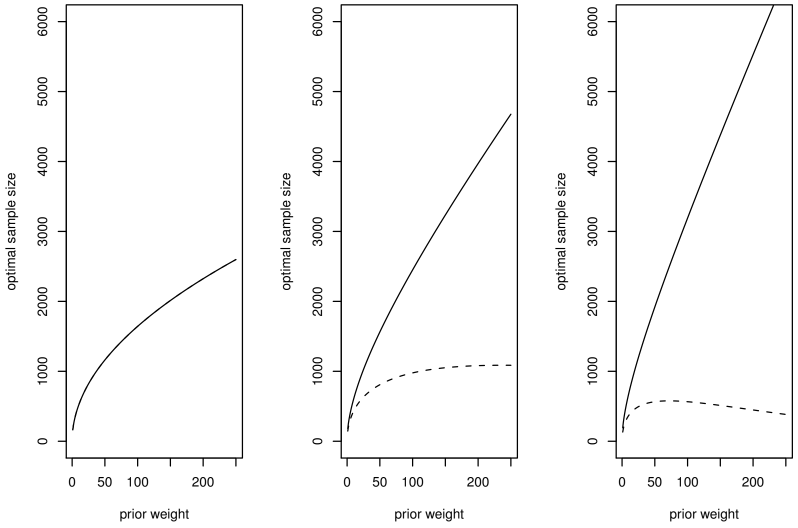

Web Figure 4: Optimal sample sizes for treatment group 1 (solid line) and treatment group 2 (dashed line) for two arm Poisson example with $h_i(\xi_i) = g_i(\xi_i) = -\xi_i$ and $N = 10800$ for varying weights, $n_{01} = n_{02}$, when the prior mean for treatment group 1 is 0.05 and the prior mean for treatment group 2 is 0.05 (left panel), 0.075 (centre panel) or 0.1 (right panel).

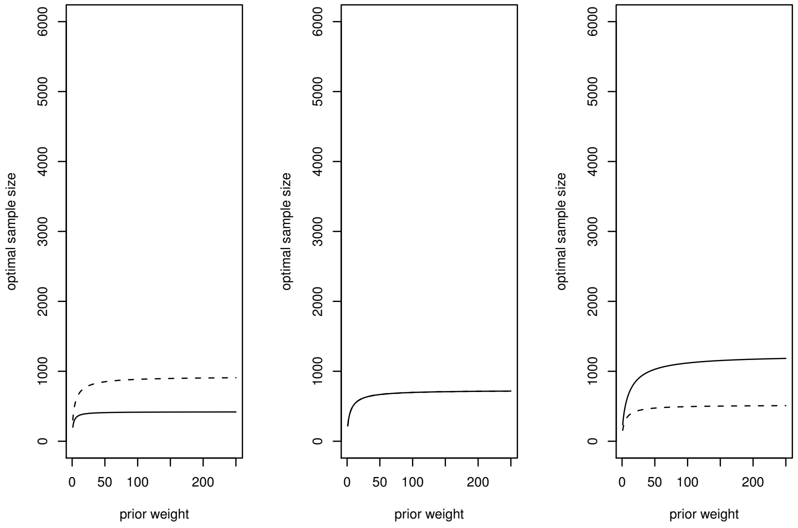

Web Figure 5: Optimal sample sizes for treatment group 1 (solid line) and treatment group 2 (dashed line) for two arm Poisson example with $h_i(\xi_i) = g_i(\xi_i) = -\xi_i$ and $N = 10800$ for varying weights n_{01}, with $n_{02} = 10$, when the prior means for treatment groups 1 and 2 are respectively 0.1 and 0.05 (left panel), 0.05 and 0.05 (centre panel) or 0.05 and 0.1 (right panel).
